# Significant response to toripalimab plus axitinib for metastatic chromophobe renal cell carcinoma with sarcomatoid differentiation: a case report and literature review

**DOI:** 10.3389/fonc.2025.1606414

**Published:** 2025-08-11

**Authors:** Xin Chen, Gonglin Tang, Weicheng Sun, Chengyue Liu, JunDong Zhao, Hongwei Zhao

**Affiliations:** ^1^ Qingdao University Medical College Affiliated Yantai Yuhuangding Hospital, Yantai, Shandong, China; ^2^ Second Clinical Medical College, Binzhou Medical University, Yantai, China; ^3^ School of Clinical Medicine, Shandong Second Medical University, Weifang, China

**Keywords:** non-clear renal cell carcinoma, sarcomatoid differentiation, immune checkpoint inhibitor, toripalimab, axitinib

## Abstract

Chromophobe renal cell carcinoma (chRCC) is a rare subtype of renal cell carcinoma (RCC). Sarcomatoid differentiation is considered a result of dedifferentiation of the primary tumor. The coexistence of both components (chromophobe and sarcomatoid) in a single renal tumor has been infrequently reported. Currently, only isolated cases of metastatic sarcomatoid chRCC treated with immune checkpoint inhibitors (ICIs) or targeted therapies have been documented. Here, we present the first case of metastatic sarcomatoid chRCC treated with toripalimab plus axitinib and provide a review of the relevant literature. During a routine physical examination, a 58-year-old male was found to have a left renal mass. Subsequent imaging with computed tomography urography (CTU) revealed a 3.4 × 2.3 cm hypovascular tumor located in the middle pole of the left kidney, which was clinically diagnosed as left renal cell carcinoma. The patient underwent robotic-assisted laparoscopic left radical nephrectomy, and histological analysis confirmed the presence of chromophobe renal cell carcinoma with sarcomatoid differentiation. Unfortunately, twelve months post-surgery, the patient was diagnosed with retroperitoneal lymph node metastasis. Treatment with toripalimab (3mg/kg, every 2weeks) plus axitinib (5 mg orally twice daily with a 12-hour interval) was initiated, and a significant response was observed after eight months. The treatment was well tolerated, with no significant adverse reactions, and the patient is currently continuing the treatment. Metastatic chromophobe renal cell carcinoma with sarcomatoid differentiation is an extremely rare occurrence. The combination of toripalimab and axitinib may represent a promising treatment option for patients with chRCC and sarcomatoid differentiation.

## Background

ChRCC represents the third most prevalent histologic subtype of renal cell carcinoma, constituting approximately 5% of all primary renal malignancies (1). Distinct from other kidney cancers, which typically arise from more proximal structures, the pathogenesis of chRCC suggests that this subtype originates from the distFal convoluted tubules (2). ChRCC demonstrates a more favorable prognostic profile with reduced metastatic propensity compared to clear-cell RCC.

Sarcomatoid differentiation is a rare feature that can occur in most histological subtypes of RCC and is associated with a notably poor prognosis (3). Only 1 to 2% of chRCC cases exhibit sarcomatoid differentiation, significantly fewer than those seen in clear-cell RCC (4, 5). While chRCC typically has a good prognosis, the presence of sarcomatoid differentiation transforms the disease biology, resulting in a high-grade, aggressive phenotype with poorer outcomes (6).

Currently, there are no reliable data regarding the clinical efficacy of systemic therapy agents in advanced chRCC with sarcomatoid differentiation, and only isolated cases involving ICIs or targeted therapy have been reported. Here, we present the first case of a patient with chRCC with sarcomatoid differentiation who demonstrated a positive response to toripalimab combined with axitinib.

## Case presentation

**Figure 1:**
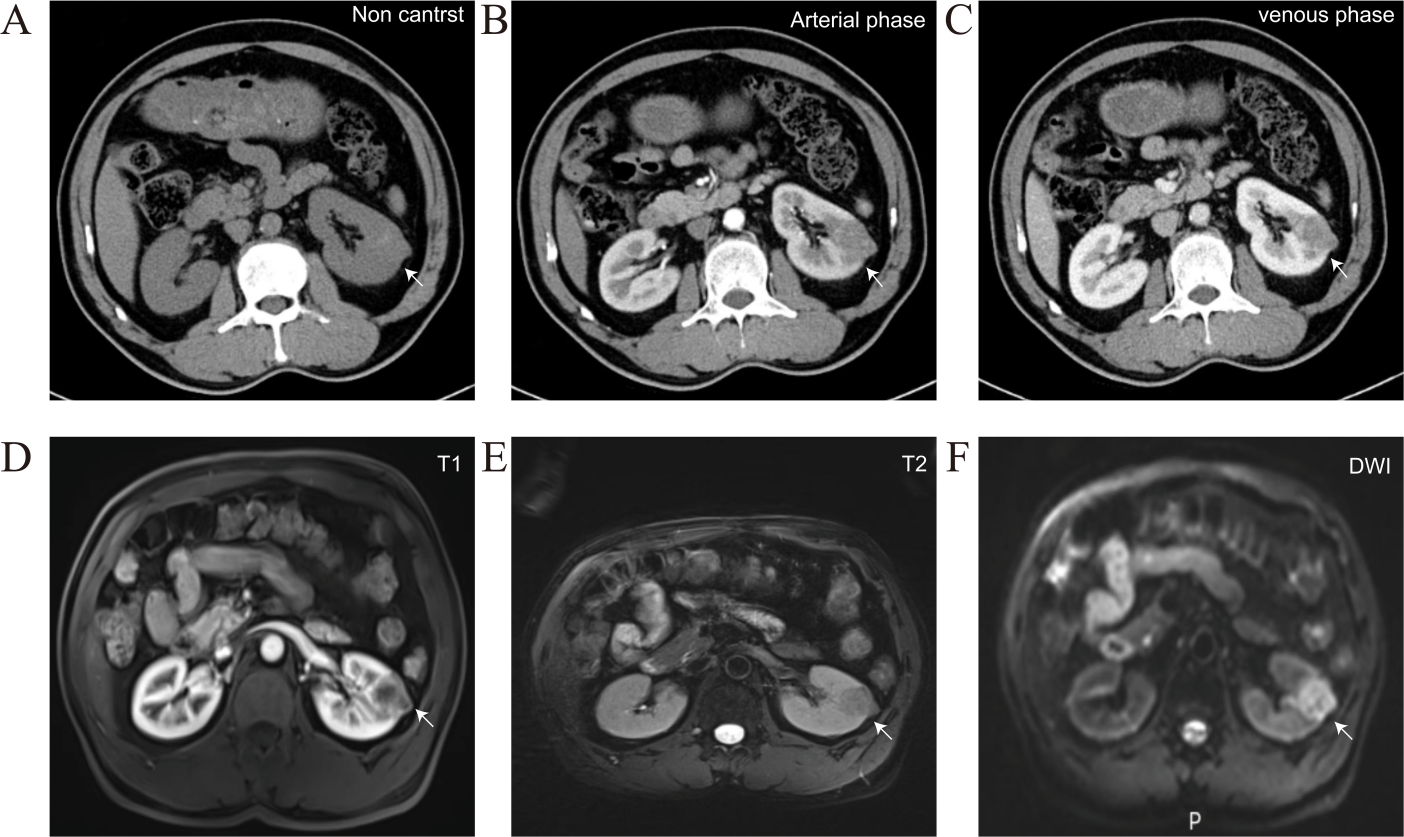
Representative CTU and MRI images of the patients, arrows indicate the location of the tumor. **(A)** Non contrast CTU scan showed irregular low-density shadow with punctate high-density foci, and its boundary was unclear. **(B, C)** Arterial phase and venous phase CTU scan showed irregular and inhomogeneous enhancement. **(D-F)** MRI scan confirmed a hypovascular tumor in the left kidney with heterogeneous delayed-phase enhancement.

**Figure 2:**
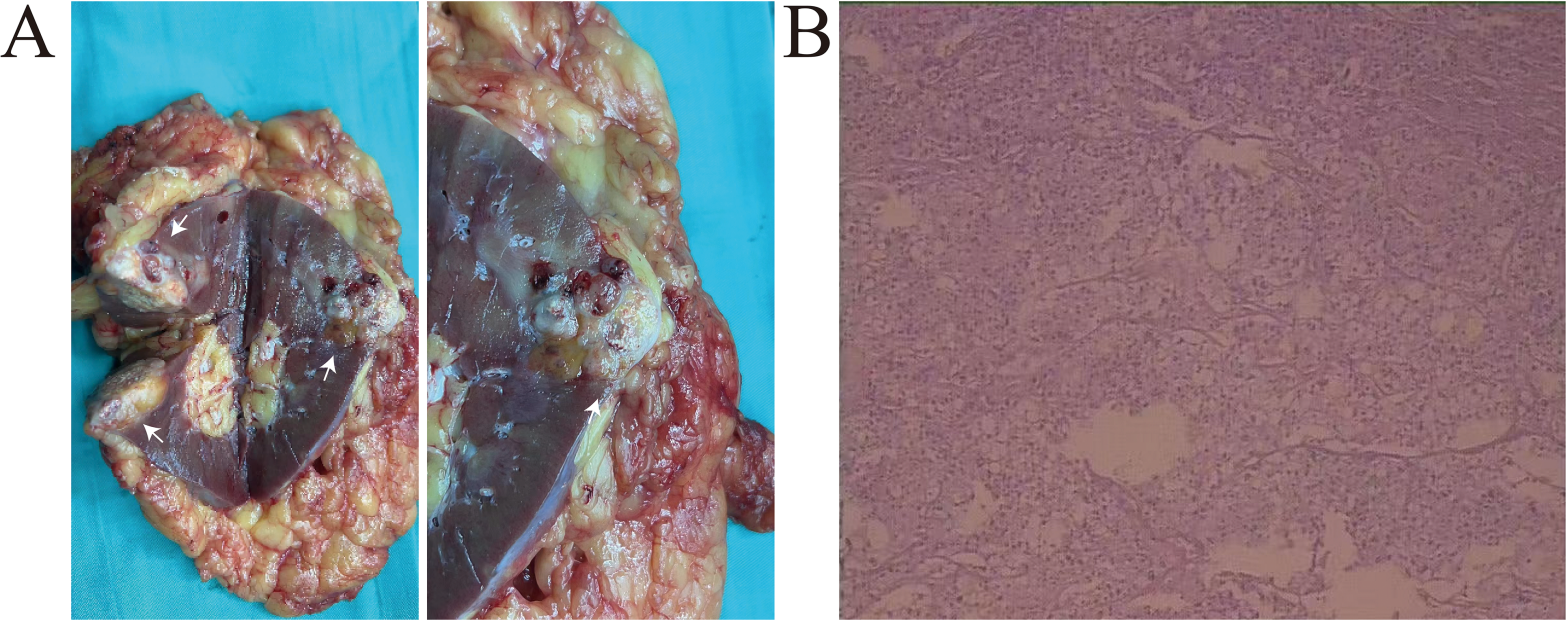
**(A)** The left kidney was completely resected, white arrows indicate the location of the tumor. The cut surface of the tumor appeared grayish-yellow in color, with areas of focal hemorrhage, necrosis, and calcification. **(B)** Histological analysis revealed two distinct morphologic components of the tumor. The chRCC region is composed of polygonal cell and the sarcomatoid component consisted spindle cells.

A 58-year-old male presented to our hospital with a left renal tumor incidentally detected during a routine abdominal ultrasonography as part of a physical examination. Subsequent computed tomography urography (CTU) revealed a 3.4 × 2.3 cm hypovascular tumor in the middle pole of the left kidney. The tumor displayed irregular low-density shadow with punctate high-density foci, and its boundary was unclear (**Figure 1A**). The enhanced scan demonstrated slightly heterogeneous enhancement (**Figures 1B, C**). Further abdominal magnetic resonance imaging (MRI) confirmed a hypovascular tumor in the left kidney with heterogeneous delayed-phase enhancement (**Figures 1D-F**). No evidence of metastatic disease was found on the CT and MRI scans. The tumor was clinically diagnosed as a left RCC and was classified as cT1aN0M0 according to the TNM system. With the family’s consent, a robotic-assisted laparoscopic left radical nephrectomy was performed. The cut surface of the tumor appeared grayish-yellow in color, with areas of focal hemorrhage, necrosis, and calcification (**Figure 2A**).

Histological analysis revealed two distinct morphologic components of the tumor. The chRCC areas were arranged in a nested pattern, with polygonal cells having centrally located, round, hyperchromatic nuclei, pale granular eosinophilic cytoplasm, and faint cytoplasmic borders. The sarcomatoid component consisted of large, pleomorphic, mitotically active spindle cells, accompanied by extensive necrosis (**Figure 2B**). The chRCC cells were positive for CK7, CD117, CA-9, SDHB and PAX-8, but negative for vimentin. The sarcomatoid cells were strongly positive for vimentin. The Ki-67 index in the sarcomatoid area was approximately 40%. Based on histological and immunohistochemical findings, the tumor was diagnosed as chromophobe renal cell carcinoma with sarcomatoid differentiation and classified as pT1aN0M0.

**Figure 3:**
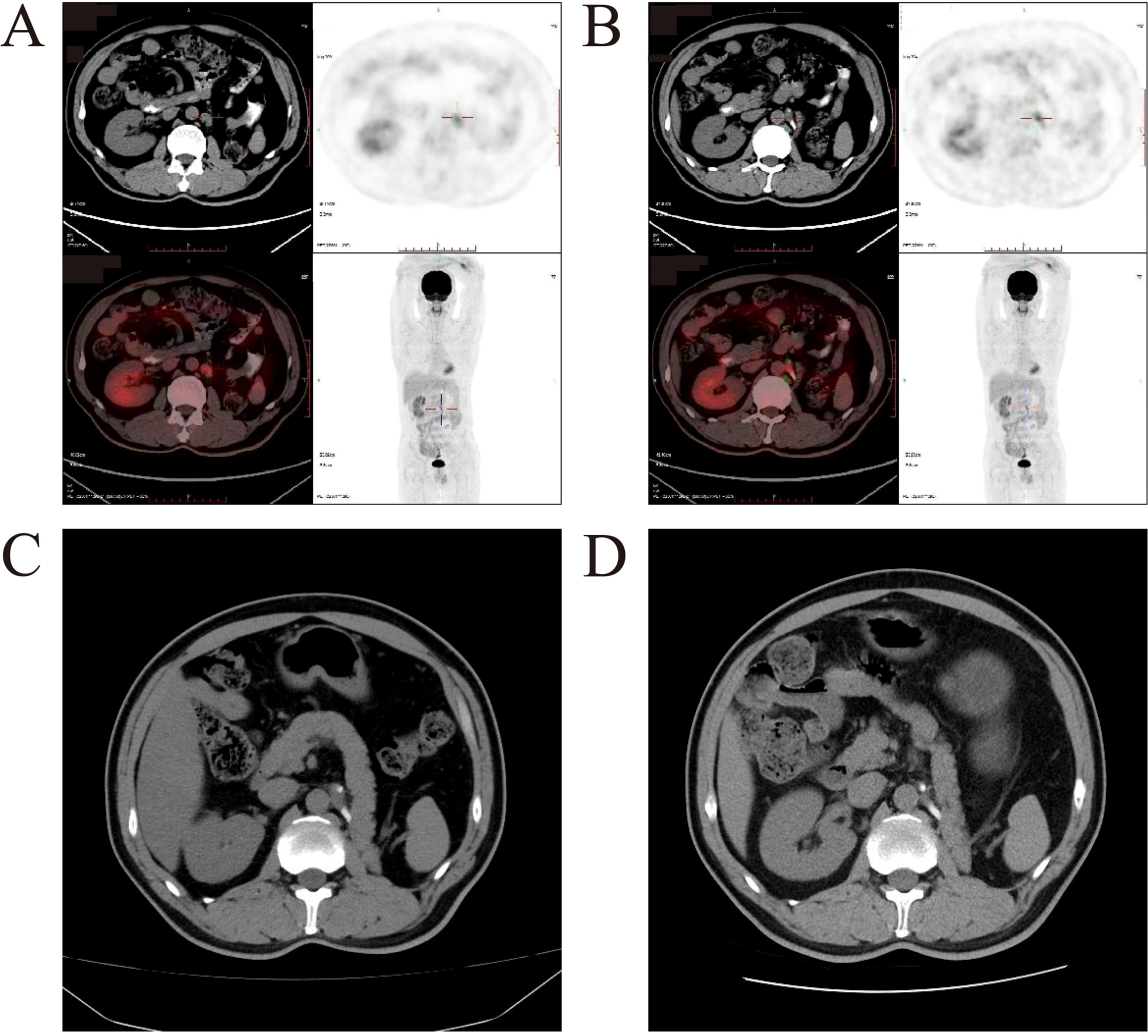
Imageological examination before and after combination therapy with toripalimab and axitinib. **(A, B)** Representative PET-CT images of patient before therapy. Enlarged lymph nodes were confirmed as tumor metastases by PET-CT. **(C)** Pre-treatment image shows retroperitoneal lymphadenopathy (white arrows). **(D)** Post-treatment image demonstrates significant reduction in the size of metastatic lymph nodes (white arrows), consistent with a partial response to therapy.

One year after the surgery, the patient developed enlarged retroperitoneal lymph nodes, and tumor metastasis was confirmed via PET-CT (**Figures 3A, B**). Treatment with axitinib and toripalimab was initiated. Following the initiation of combination therapy with toripalimab (3 mg/kg every 2 weeks) and axitinib (5 mg orally twice daily), the patient demonstrated a marked clinical and radiographic response. The patient demonstrated a favorable clinical and radiographic response. Pre-treatment imaging revealed retroperitoneal lymphadenopathy indicative of metastatic disease. Post-treatment contrast-enhanced CT scans showed significant regression of the metastatic lymph nodes (**Figures 3C, D**). The treatment was well tolerated, with no grade ≥3 adverse events observed and no immune-related adverse events occurred during therapy. To date, the patient has completed 8 months of follow-up, demonstrating sustained tolerance to the treatment regimen with no treatment-related adverse events reported. Ongoing active surveillance continues to assess long-term clinical outcomes. The patient continued the regimen without interruption. No immune-related adverse events, including thyroid dysfunction or other endocrine abnormalities, were observed during treatment or follow-up.

## Discussion

RCC comprises a spectrum of neoplastic lesions originating from the renal parenchyma, characterized by distinct histopathological features. The most prevalent subtype, clear cell renal cell carcinoma (ccRCC), accounts for approximately 80% of RCC cases. The remaining 20% are classified as non-clear cell renal cell carcinoma (nccRCC), which includes various subtypes distinguished by unique molecular and cytogenetic profiles. Among nccRCC, papillary and chromophobe subtypes constitute the predominant histologic variant (7). Studies indicate that patients diagnosed with chRCC exhibit significantly better cancer-specific survival rates compared to those with ccRCC (8).

Sarcomatoid differentiation is a histopathologic feature found in 5% to 20% of RCC tumors across different histological types and is typically associated with an aggressive phenotype (3). Only 1 to 2% of chRCC cases exhibit sarcomatoid differentiation, significantly fewer than in ccRCC (4, 5). The presence of sarcomatoid differentiation in chRCC alters the disease biology, resulting in a high-grade, aggressive phenotype with poor outcomes, including early recurrence after nephrectomy and limited effectiveness of standard targeted therapies in metastatic cases (6). The case presented herein aligns with these observations. These findings emphasize the need for further investigation into the biological mechanisms driving this variant of chRCC and the development of systematic management strategies specifically tailored to this subtype of the disease.

Toripalimab is a humanized PD-1 monoclonal antibody that prevents the interaction between PD-1 and its ligands (PD-L1 and PD-L2). It was approved by the National Medical Product Administration in China in 2018 as a second-line therapy for metastatic melanoma (9). The randomized phase III RENOTORCH trial evaluated toripalimab plus axitinib against sunitinib in patients with previously untreated advanced RCC. Patients receiving toripalimab plus axitinib showed significantly and clinically meaningful improvements in PFS and ORR compared to those receiving sunitinib (10). Although this trial is design for ccRCC, its findings underscore the potential of combining PD-1 inhibitors with VEGF-targeted agents to enhance antitumor immunity. Currently, this combination has not yet been established as a standard therapy for non-ccRCC. Given the lack of established therapies for metastatic non-ccRCC and most guidelines recommend clinical trials for metastatic non-ccRCC, we adopted this regimen based on its mechanistic rationale and emerging evidence supporting immune checkpoint inhibitors in RCC, irrespective of histologic subtype. To our knowledge, we present the first documented case of a response to toripalimab in a patient with chRCC with sarcomatoid differentiation.

The combination of chRCC with sarcomatoid differentiation is an exceptionally rare clinical entity. Due to the rarity of such cases, only a few studies have reported systemic therapies for metastatic chRCC with sarcomatoid differentiation. We reviewed these studies (**Table 1**), which suggest that metastatic chRCC with sarcomatoid differentiation may respond well to PD-1 immune checkpoint inhibitors. Rouvinov et al. published the first case report on this clinical entity, documenting a significant response to nivolumab (11). Similarly, Noguchi et al. reported a case of a patient with metastatic sarcomatoid chRCC who demonstrated a significant response to nivolumab, following unsuccessful targeted therapies such as sunitinib, pazopanib, and axitinib due to adverse events or disease progression (12). Additionally, Fukushima T et al. described a patient who, after experiencing failure with sunitinib, responded well to nivolumab, while another patient benefitted from a combination of ipilimumab and nivolumab (13). In contrast, metastatic chromophobe carcinoma with sarcomatoid differentiation seems to exhibit limited effectiveness when treated with targeted therapies. Lichtbroun et al. reported of a patient treated with targeted therapies such as sunitinib, erlotinib, and everolimus, but the disease continued to progress, indicating a lack of response to these treatments (14). Compared to targeted monotherapies, immune checkpoint inhibitor-based therapies appear to offer more clinical significance in the treatment of metastatic sarcomatoid chRCC. Therapeutic management of metastatic sarcomatoid chRCC must be individualized, accounting for key factors including patient age, pathologic subtype characteristics, and drug accessibility. In our case, the patient’s age (58 years) and preserved organ function permitted full-dose combination therapy without dose reduction. Pathologically, sarcomatoid differentiation characterized by high PD-L1 expression provides a strong rationale for prioritizing immune checkpoint inhibitors over VEGF-targeted monotherapy. For regions where toripalimab is unavailable, other PD-1/PD-L1 inhibitors (nivolumab or pembrolizumab) may be substituted with axitinib, given their shared mechanisms of action and proven efficacy in RCC.

**Table 1:** Review of systemic therapy for metastatic sarcomatoid chRCC.

No.	Study	Age/Gender	Tumor size	Surgical stage	Metastasis	Systemic therapy Options	Outcomes
Case 1	Lichtbroun BJ et al. (14)	47/male	18 cm	T4N0	Retroperitoneal masses, lung	Sunitinib, erlotinib, everolimus	Disease continued to progress, died 15 months after surgery.
Case 2	Lichtbroun BJ et al. (14)	63/male	26 cm	T4N0	Peritoneal mass	Nivolumab	Died 5 weeks after starting nivolumab and 161 days after surgery.
Case 3	Lichtbroun BJ et al. (14)	51/female	15 cm	T3aN1	Retroperitoneal lymph node, left psoas mass, neck and mediastinum	Pembrolizumab plus axitinib, cabozantinib	Disease worsening after 2 years starting pembrolizumab plus axitinib, treatment change to cabozantinib, at last follow-up, the patient is 44 months from surgery.
Case 4	Fukushima T et al. (13)	63/male	10×13×11 cm	NA	Celiac lymph node, liver, lung	Sunitinib, nivolumab	Disease progression after a month starting sunitinib, treatment change to nivolumab and discontinued after 9 cycles, the metastatic lesions continued to shrink over the next 3.5 years without any agent.
Case 5	Fukushima T et al. (13)	74/male	5.9×5.7 cm	NA	Right lung and bronchial, left 8th rib	Ipilimumab plus nivolumab	The metastatic lesions continued to shrink at 4 months, while all metastatic lesions remained at 6 months.
Case 6	Noguchi G et al. (12)	41/female	9.5 cm	T2bN0M0	Multiple lung masses	Sunitinib, pazopanib, everolimus, sorafenib, axtinib, temsirolimus, nivolumab	Drug eruption or disease progression after treatment with sunitinib, pazopanib, everolimus, sorafenib, axtinib and temsirolimus. Significant clinical improvement was noted after 12 cycles nivolumab.
Case 7	Rouvinov K et al. (11)	52/male	3.5 cm	NA	Retroperitoneal mass	Nivolumab	After 6 cycles a partial response was seen and the treatment was well tolerated.
Case 8	Matrana MR et al. (26)	82/ female	NA	NA	Pancreas, lung,	Pazopanib, sorafenib	The patient received pazopanib at full dose for 8 months, until she developed progressive disease. She then received sorafenib for 2 months with progressive disease.

The prognosis for chRCC patients is generally more favorable than for other RCC types, with fewer patients presenting with metastatic disease. However, due to the rarity of metastatic chRCC, prospective clinical trials are often lacking. While therapeutic strategies for metastatic ccRCC have advanced significantly over the past decade, established treatment guidelines for chRCC remain inadequately defined. In a randomized multicenter phase II trial comparing sunitinib and everolimus in patients with nccRCC, including 12 individuals with chRCC, six patients who received sunitinib had a median PFS of 8.9 months (15). Another phase II trial involving 16 patients with chRCC showed that everolimus was associated with a longer median PFS compared to sunitinib (16). A phase II trial by Lee et al. evaluated the combination of cabozantinib and nivolumab in patients with nccRCC, showing promising efficacy in most nccRCC variants, but limited treatment effects in chRCC (17). The KEYNOTE-427 trial assessed the efficacy and safety of pembrolizumab monotherapy in 165 patients with treatment-naive advanced nccRCC. The results indicated that pembrolizumab was less effective in chRCC compared to other nccRCC subtypes (18).

Sarcomatoid differentiation is not a true histological classification and can be observed in any RCC histology. Sarcomatoid RCC is associated with a poor prognosis and limited responsiveness to anti-angiogenic targeted therapies but has shown particularly favorable responses to ICIs (19–21). Tumor expression of PD-L1 is associated with improved responses to PD-1 and PD-L1 blocking agents. This favorable efficacy to immune checkpoint inhibitors may be linked to an enhanced immunogenic profile and an increased expression of PD-L1 observed in patients with a sarcomatoid component (22). The constitutive patterns of PD-L1 expression found in a subset of patients with sarcomatoid RCC, where every tumor cell exhibits high levels of PD-L1 expression (23). Additionally, RCC tumors with prominent sarcomatoid differentiation are often characterized by a high Ki-67 index and the absence of Von Hippel-Lindau gene mutations (24). These findings contribute to the growing understanding of the immune microenvironment in sarcomatoid RCC tumors, highlighting the need for further research on the potential advantages of PD-1 and PD-L1 immune checkpoint blockade therapy. The response to toripalimab in this sarcomatoid chRCC is mechanistically supported by the immunogenic nature of sarcomatoid differentiation. First, sarcomatoid transformation induces constitutive PD-L1 overexpression on tumor cells and tumor-infiltrating immune cells, creating a microenvironment primed for PD-1 blockade. Toripalimab, as a humanized anti-PD-1 monoclonal antibody, binds PD-1 receptors on T cells, reversing T-cell exhaustion and restoring antitumor cytotoxicity. These mechanisms collectively enable robust immunologic clearance of tumor cells upon PD-1 inhibition.

Several limitations exist in the present study. First, PD-L1 expression testing was not performed on the tumor tissue. While PD-L1 status is often used to guide immune checkpoint inhibitor therapy, current clinical evidence suggests that PD-1 inhibitors may demonstrate efficacy in renal cell carcinoma irrespective of PD-L1 expression levels. Besides, studies have pointed out that the predictive role of PD- L1 has become less clear as combinations of ICIs with other Anticancer drugs (25). Second, the 8-month follow-up period remains insufficient to comprehensively evaluate long-term clinical outcomes, including durability of response, risk of disease recurrence, and potential late-onset treatment-related adverse events. While the observed partial response and tolerability are promising, larger cohorts and prolonged follow-up are warranted to validate these preliminary results.

## Conclusions

Metastatic chRCC with sarcomatoid differentiation is a rare and historically challenging entity with a poor prognosis. We report the first case of metastatic chRCC with sarcomatoid differentiation treated with toripalimab plus axitinib. PD-L1 expression is significantly enriched in renal cell carcinomas with sarcomatoid differentiation. This case suggests that ICIs-based therapy may have a clinically meaningful effect on these tumors. Therefore, ICIs-based therapy should be considered a preferred treatment option for patients with chRCC exhibiting sarcomatoid features.

## Data Availability

The original contributions presented in the study are included in the article/Supplementary Material. Further inquiries can be directed to the corresponding author.
